# Supplementary material for: Targeting granule initiation and amyloplast structure to create giant starch granules in wheat
Source: Sci Adv. 2026 Jul 3;12(27):eaeh2735. doi: 10.1126/sciadv.aeh2735 (PMC13330829; doi:10.1126/sciadv.aeh2735)
Supplement: Supplementary file 1 — Figs. S1 to S9 Tables S1 and S2 Legend for data file S1 [file sciadv.aeh2735_sm.pdf]

Science Advances
AAAS

Supplementary Materials for

Targeting granule initiation and amyloplast structure to create giant starch granules in wheat

Rose McNelly *et al.*

Corresponding author: Rose McNelly, rose.mcnelly@jic.ac.uk; David Seung, david.seung@jic.ac.uk

Sci. Adv. **12**, eaeh2735 (2026)
DOI: 10.1126/sciadv.aeh2735

The PDF file includes:

Figs. S1 to S9
Tables S1 and S2
Legend for data file S1

Other Supplementary Material for this manuscript includes the following:

Data file S1

Fig. S1.

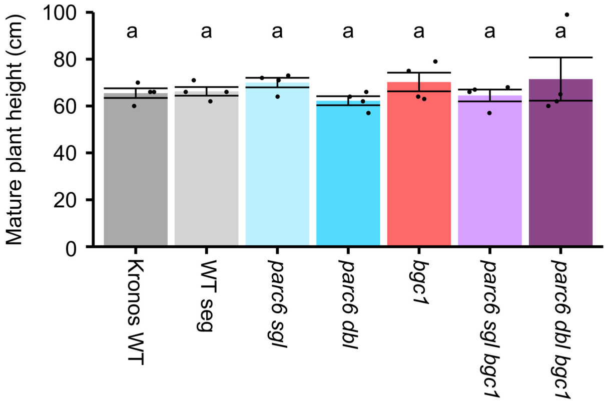

There is no difference in the height of mature *parc6 bgc1* mutants. Data are presented as means ± standard error of the mean, with individual data points shown as black dots. Values with different letters are significantly different under a one-way ANOVA and Tukey's post hoc test at $P < 0.05$ ($N = 4$ per genotype).

Fig. S2.

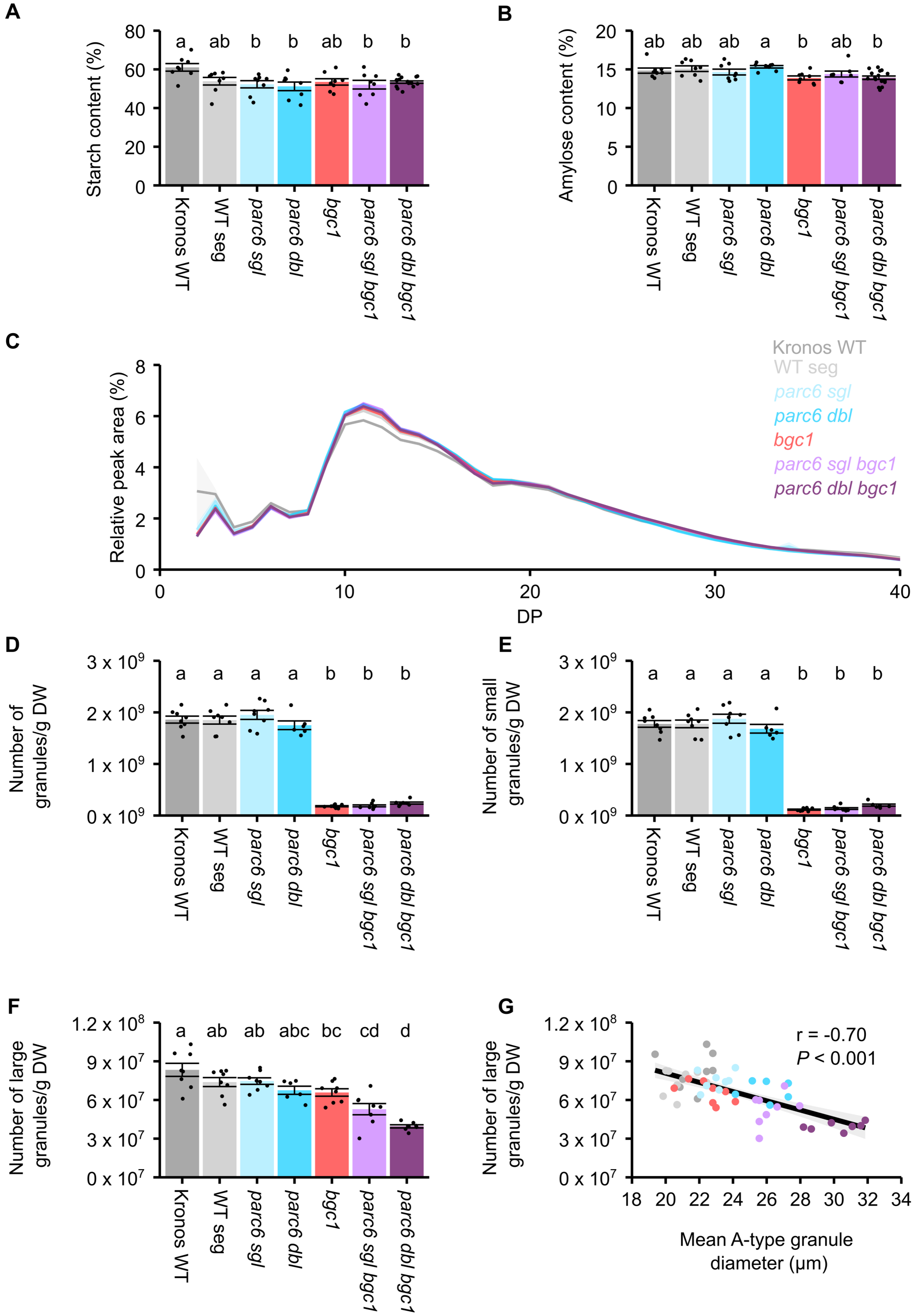

Amylose content, total starch content and granule number in *parc6 bgc1* mutants. (A) Total starch content of wholewheat flour. (B) Amylose content of purified starch. Data in (A)-(B) are presented as means ± standard error of the mean, with individual data points shown as black dots. Values with different letters are significantly different under a one-way ANOVA and Tukey's post hoc test at $P < 0.05$ ($N = 8$-16 per genotype). (C) Chain length distribution of

purified starch, data are represented as means (solid lines) ± standard error of the mean (shading), ($N = 3$-4 per genotype). (D) Starch granule number in mature grains. Starch was purified, and the number of granules was determined using a Coulter counter running in volumetric mode. Values are expressed relative to the dry weight of the grain. (E) Starch granule number from (D) but only granules <10 µm were counted. (F) Starch granule number from (D) but only granules >10 µm were counted. In (D-F), data are presented as means ± standard error of the mean, with individual data points shown as black dots. Values with different letters are significantly different under a one-way ANOVA and Tukey’s post hoc test at $P < 0.05$ ($N = 6$-8 per genotype). (G) The number of large granules (F) correlated against mean A-type granule diameter (from Fig 2d). The black line represents a linear model between the parameters with the grey shading representing 95% confidence intervals. A Pearson correlation test was conducted and the correlation coefficient (r) and *P* value are on the top right of (g).

Fig. S3.

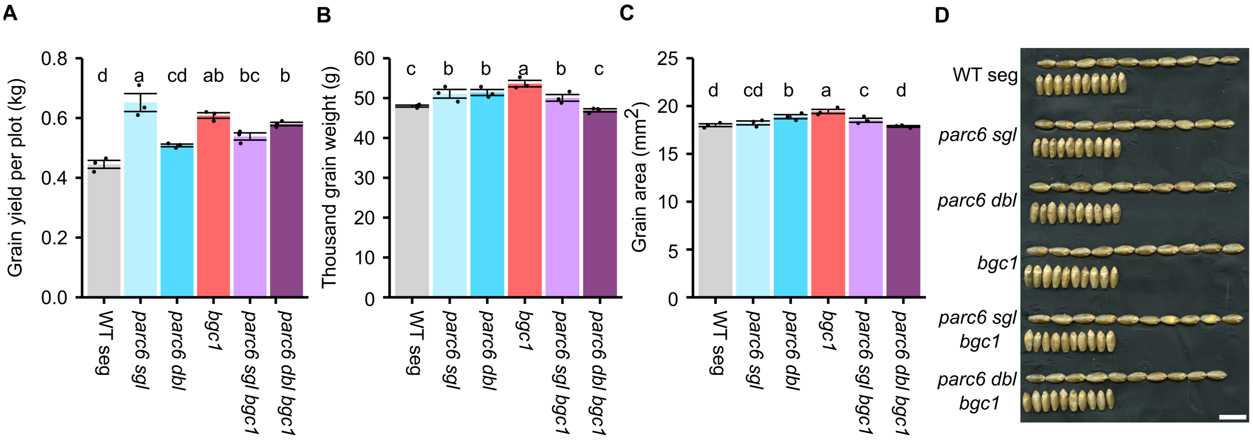

Grain phenotypes of field-grown *parc6 bgc1* mutants. (A) Grain yield per 1m^2 plot. (B) Thousand grain weight. (C) Grain size as measured by 2D grain area. (D) Photographs of grains showing both the dorsal and ventral sides. Bar = 1 cm. In (A), (B) and (C) data are presented as means ± standard error of the mean, with individual data points shown as black dots. Values with different letters are significantly different under a one-way ANOVA and Tukey's post hoc test at $P < 0.05$ ($N = 3$ per genotype).

Fig. S4.

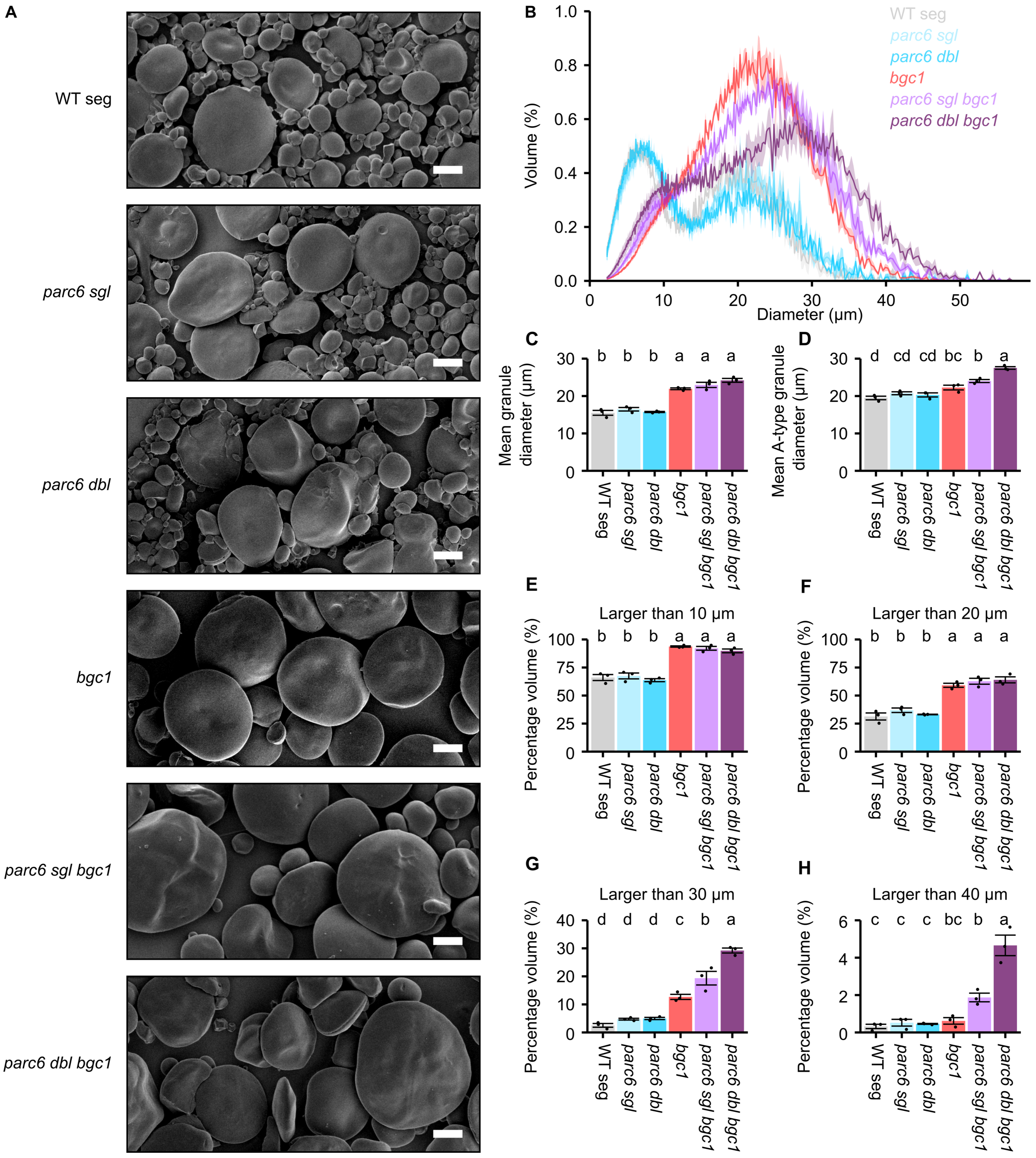

Granule size phenotypes are reproducible in field-grown *parc6 bgc1* mutants. (A) Scanning electron microscopy of purified starch. Bar = 10 µm. (B) Starch was purified from mature grains and a Coulter counter was used to measure size distribution traces. The traces are displayed as means (solid lines) ± standard error of the mean (shading) and have been adjusted for representation on a linear *x* scale. (C) Mean granule diameter, this accounts for both A-type and B-type granules. (D) Mean granule diameter of A-type granules only. (E-H) The percentage (by volume) of granules larger than: (E) 10 µm, (F) 20 µm, (G) 30 µm, (H) 40 µm. Data are presented as means ± standard error of the mean, with individual data points shown as black

dots, note the altered y-axis scale in (G) and (H) which has been adjusted for clarity. Values with different letters are significantly different under a one-way ANOVA and Tukey's post hoc test at $P < 0.05$ ($N = 3$ per genotype).

Fig. S5.

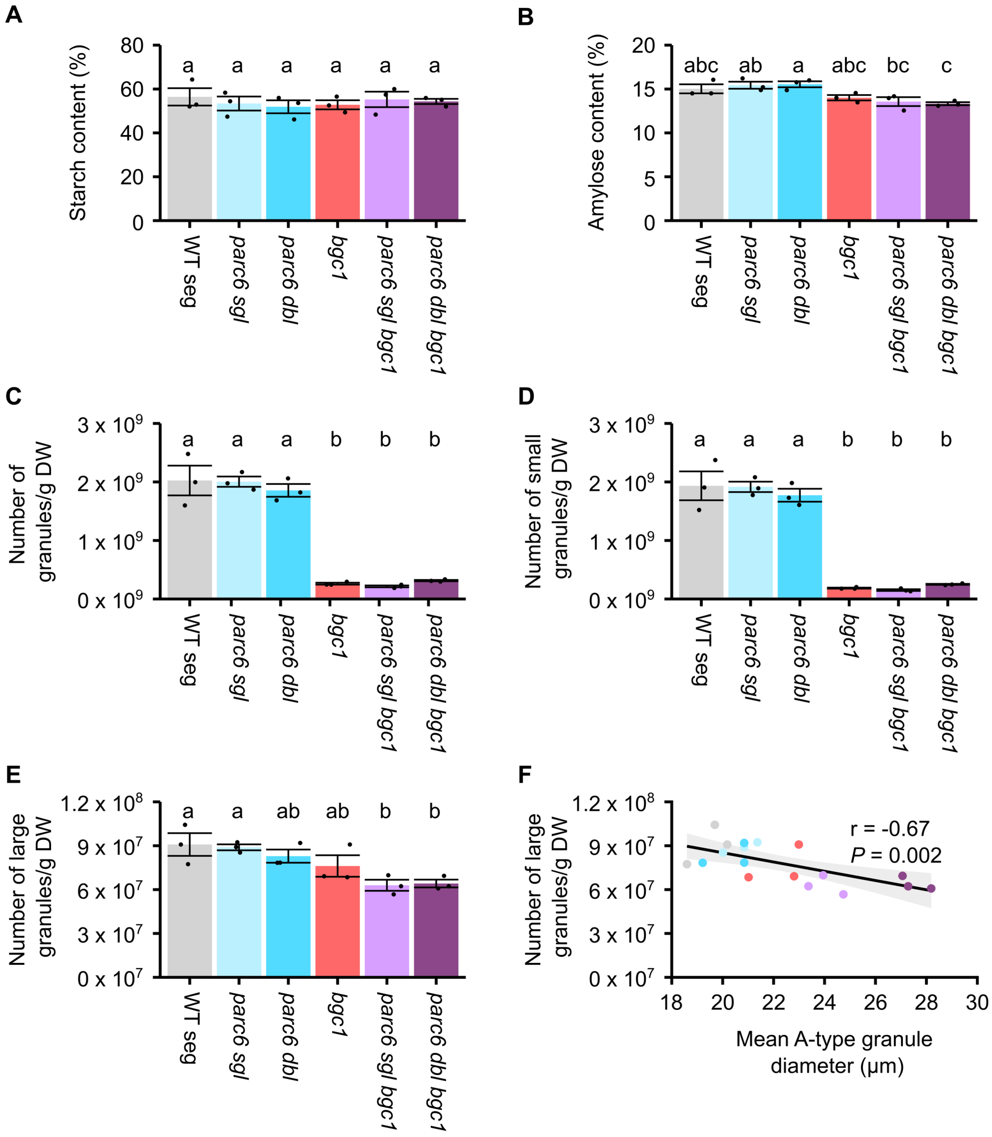

Amylose content, total starch content and granule number in field-grown *parc6 bgc1* mutants. (A) Amylose content of purified starch. (B) Total starch content of wholewheat flour. Values with different letters are significantly different under a one-way ANOVA and Tukey's post hoc test at $P < 0.05$ ($N = 3$ per genotype). (C) Starch granule number in mature grains. Starch was purified, and the number of granules was determined using a Coulter counter running in volumetric mode. Values are expressed relative to the dry weight of the grain. (D) Starch granule number from (C) but only granules <10 µm were counted. (E) Starch granule number from (C) but only granules >10 µm were counted. In (A-E), data are presented as means ± standard error of the mean, with individual data points shown as black dots. Values with different letters are significantly different under a one-way ANOVA and Tukey's post hoc test at $P < 0.05$ ($N = 3$ per genotype). (F) The number of large granules (E) correlated against mean A-type granule diameter (from Fig S4D). The black line represents a linear model between the

parameters with the grey shading representing 95% confidence intervals. A Pearson correlation test was conducted and the correlation coefficient (r) and *P* value are shown.

Fig. S6.

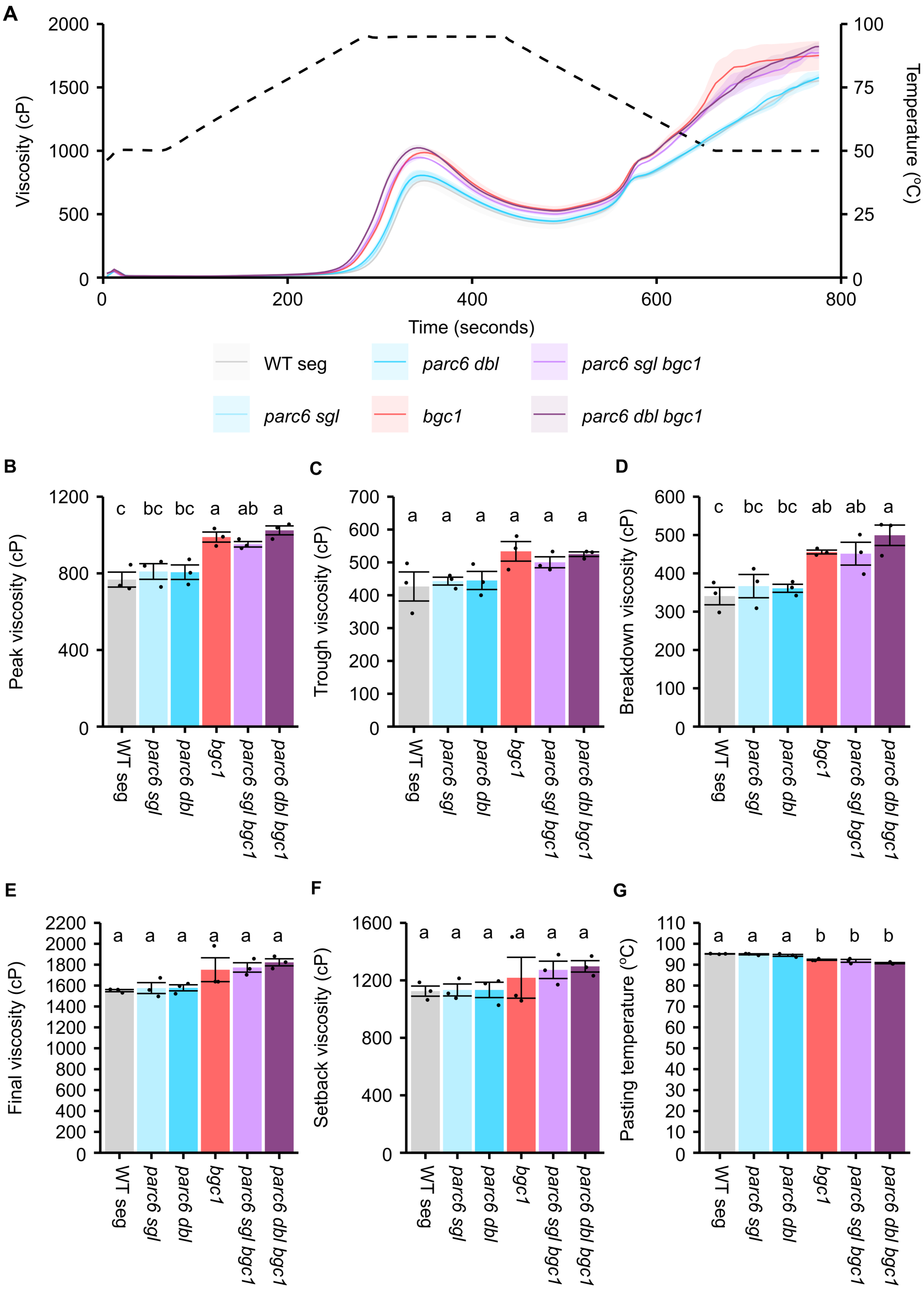

The *parc6 bgc1* mutants have increased peak and breakdown viscosities. (A) Rapid Visco Analyser (RVA) analysis of viscosity of purified starch (2 g in 25 mL water) during gelatinisation. Data are presented as means (solid line) ± SEM (shading) of three biological

replicates, each using starch from grain harvested from a different field plot. Temperature changes are displayed as a dotted line on the right hand axis. Parameters from the viscographs in (A) were obtained. (B) Peak viscosity, (C) trough viscosity, (D) breakdown viscosity, (E) final viscosity, (F) setback viscosity, (G) pasting temperature. Data are presented as means ± standard error of the mean, with individual data points shown as black dots. Values with different letters are significantly different under a one-way ANOVA and Tukey's post hoc test at $P < 0.05$ ($N = 3$ per genotype).

Fig. S7.

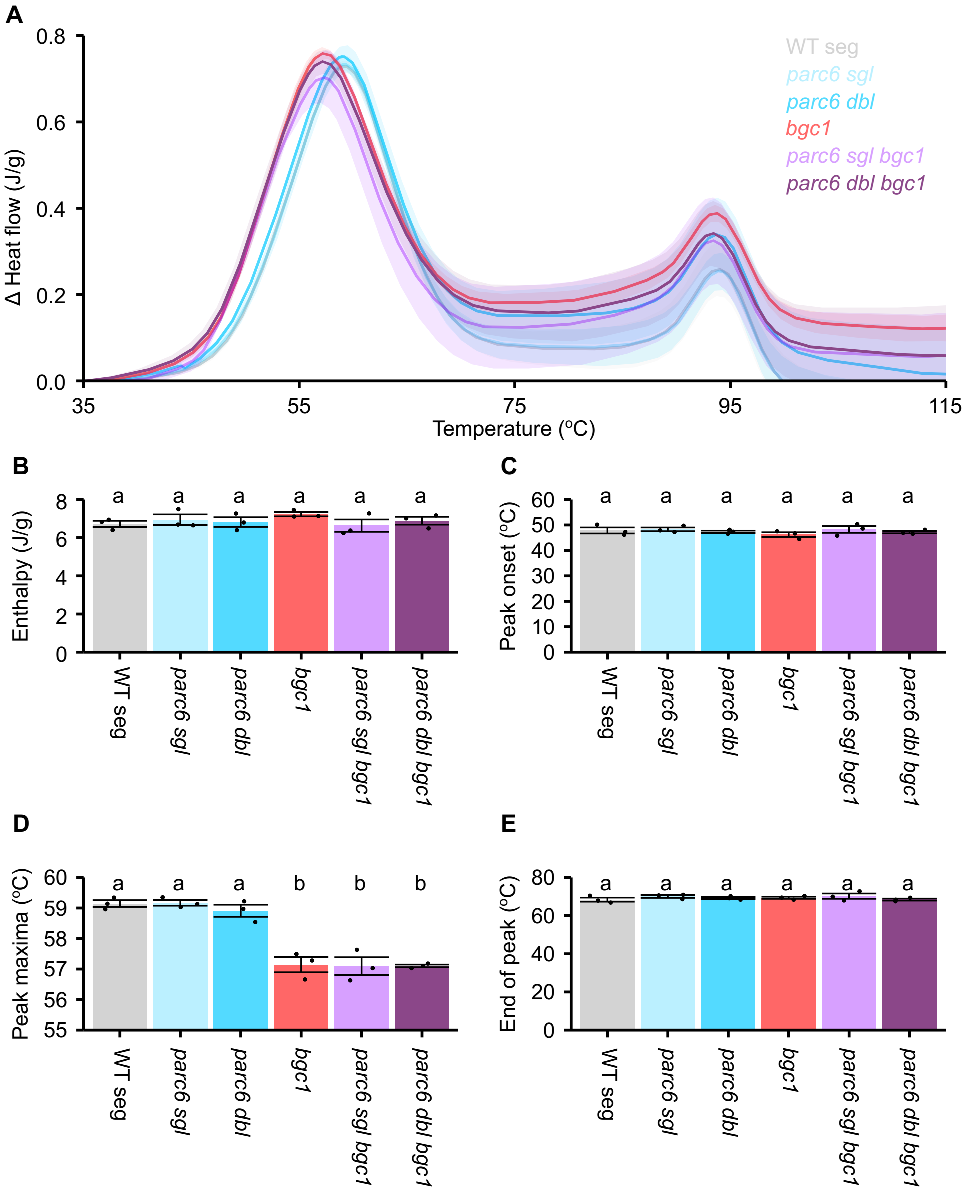

The *bgc1* starch has a shifted gelatinisation peak in Differential Scanning Calorimetry (DSC). (A) Purified starch (100 mg) was analysed on a DSC heating from 10°C to 150°C. The baseline heat flow was subtracted and data are presented as means (solid line) ± SEM (shading) of three biological replicates, each using starch from grain harvested from a different field plot. Only data from 35°C to 115°C are shown as this is where the peaks occur. The first peak was analysed - (B) enthalpy, (C) peak onset, (D) peak maxima, (E) end of peak. Data are presented as means ± SEM, with individual data points shown as black dots. Values with different letters are significantly different under a one-way ANOVA and Tukey's post hoc test at $P < 0.05$ ($N = 3$ per genotype).

Fig. S8.

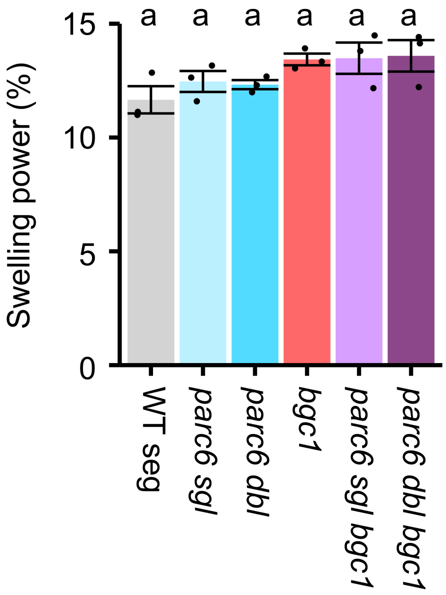

Starch from *parc6 bgc1* lines have no difference in swelling power. Swelling power of purified starch (10 mg) at 80ºC. Data are presented as means ± SEM, with individual data points shown as black dots. Values with different letters are significantly different under a one-way ANOVA and Tukey's post hoc test at $P < 0.05$ ($N = 3$ per genotype).

Fig. S9.

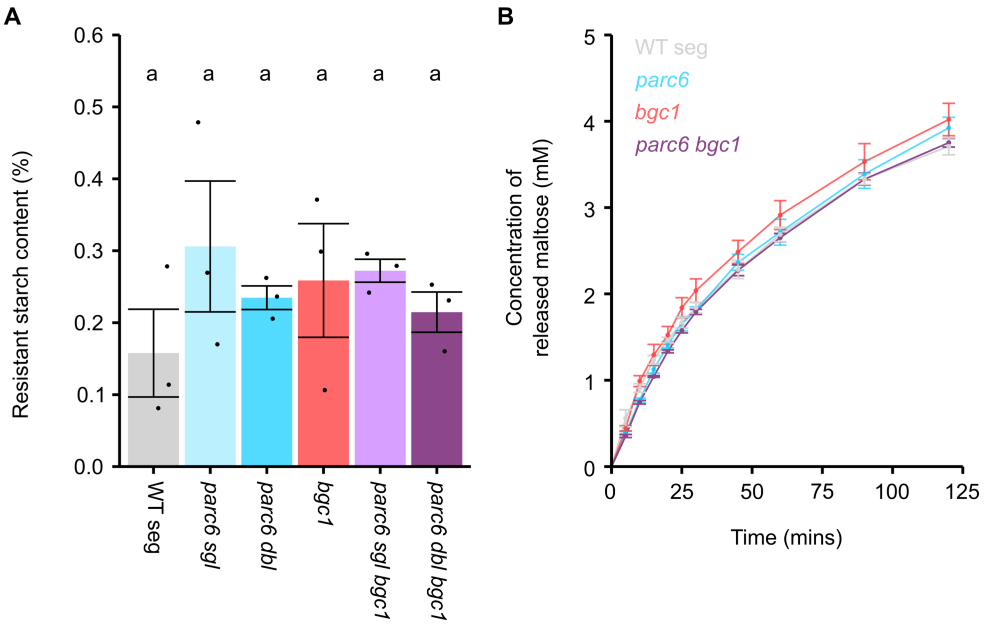

Purified *parc6 bgc1* starches have no differences in *in vitro* digestibility. (A) Resistant starch content of wholemeal flour. Data are presented as means ± SEM, with individual data points shown as black dots. Values with different letters are significantly different under a one-way ANOVA and Tukey's post hoc test at $P < 0.05$. (B) Digestion of purified starch (100 mg) from WT seg, *parc6 dbl*, *bgc1* and *parc6 dbl bgc1* with pancreatic α-amylase. Data are presented as means ± SEM with values from three independent experiments, each using starch from grain harvested from a different field plot ($N = 3$ per genotype).

Table S1.

**Name used throughout this study**	**Genotype at the *PARC6* loci**	**Genotype at the *BGC1* loci**	**Previous studies using mutants**
Kronos WT	AABB	AABB	-
WT seg	AABB	AABB	-
parc6 sgl	*aa*BB	AABB	Esch et al. 2023 (14)
parc6 dbl	*aabb*	AABB	Esch et al. 2023 (14)
bgc1	AABB	*aabb*	Chia et al. 2020 (19)
parc6 sgl bgc1	*aa*BB	*aabb*	-
parc6 dbl bgc1	*aabb*	*aabb*	-

Summary of mutants used in this study. AA refers to wild-type in the A genome, BB refers to wild-type in the B genome, *aa* refers to mutant in the A genome, *bb* refers to mutant in the B genome.

Table S2.

Locus	Line	Primers
PARC6-A1	Kronos1265	WT: gaaggtcggagtcaacggattcgagaagagtccttttgagctctc<br>Mutant: gaaggtgaccaagttcatgctcgagaagagtccttttgagctctt<br>Common: gcctatccgttgatccctggc
PARC6-B1	Kronos2369	WT: gaaggtcggagtcaacggatttgcaacataccagtgactg<br>Mutant: gaaggtgaccaagttcatgcttgcaacataccagtgacta<br>Common: cagcttcaaagtaatggaagttccaattcaaga
BGC1-A1	Kronos2244	WT: gaaggtcggagtcaacggattttgtcaagagaccatgttcgc<br>Mutant: gaaggtgaccaagttcatgctttgtcaagagaccatgttcgt<br>Common: ctcctagtcgaagtccaacta
BGC1-B1	Kronos3239	WT: gaaggtcggagtcaacggattgcccaatcctgcttcagaag<br>Mutant: gaaggtgaccaagttcatgctgcccaatcctgcttcagaaa<br>Common: gccacccattttgtagttagttag

Primers used for KASP genotyping.

Data File S1. Data spreadsheet. These spreadsheets contain data used to produce Figures 1-4 and S1-S9.
